# Sensitivity-Enhancing Modified Holographic Photopolymer of PQ/PMMA

**DOI:** 10.3390/polym16111484

**Published:** 2024-05-23

**Authors:** Junhui Wu, Junchao Jin, Po Hu, Jinhong Li, Zeyi Zeng, Qingdong Li, Jie Liu, Mingyong Chen, Zuoyu Zhang, Li Wang, Xiao Lin, Xiaodi Tan

**Affiliations:** 1 College of Photonic and Electronic Engineering, Fujian Normal University, Fuzhou 350117, China; junhuifjnu@foxmail.com (J.W.); qbx20210097@yjs.fjnu.edu.cn (J.J.); ljh0401_fjnu@163.com (J.L.); zzy947633474@foxmail.com (Z.Z.); lee1735512161@foxmail.com (Q.L.); liujie_2120@163.com (J.L.); mingychen@foxmail.com (M.C.); fjunnin9@163.com (Z.Z.); li18937012859@163.com (L.W.); 2 Henan Provincial Key Laboratory of Intelligent Lighting, Huanghuai University, Zhumadian 463000, China; hupo_lzu_13@163.com; 3 Information Photonics Research Center, Key Laboratory of Optoelectronic Science and for Medicine of Ministry of Education, Fujian Provincial Key Laboratory of Photonics Technology, Fujian Provincial Engineering Technology Research Center of Photoelectric Sensing Application, Fujian Normal University, Fuzhou 350117, China

**Keywords:** photopolymer, dendritic crosslinker, holographic storage, high photosensitivity, quantum chemical analysis

## Abstract

Phenanthrenequinone-doped poly(methyl methacrylate) (PQ/PMMA) photopolymers are potential holographic storage media owing to their high-density storage capacities, low costs, high stability, and negligible shrinkage in volume holographic permanent memory. However, because of the limitations of the substrate, conventional Plexiglas materials do not exhibit a good performance in terms of photosensitivity and molding. In this study, the crosslinked structure of PMMA was modified by introducing a dendrimer monomer, pentaerythritol tetraacrylate (PETA), which increases the photosensitivity of the material 2 times (from ~0.58 cm/J to ~1.18 cm/J), and the diffraction efficiency is increased 1.6 times (from ~50% to ~80%). In addition, the modified material has a superior ability to mold compared to conventional materials. Moreover, the holographic performance enhancement was evaluated in conjunction with a quantum chemical analysis. The doping of PETA resulted in an overall decrease in the energy required for the reaction system of the material, and the activation energy decreased by ~0.5 KJ/mol in the photoreaction stage.

## 1. Introduction

Photopolymers have been garnered considerable interest owing to their low costs and easy fabrication. They can be used in high-tech fields such as 3D display, security, anticounterfeiting, and data storage [1,2,3], etc. In response to the exploding need for data storage, the environmentally friendly optical data storage is a good choice for the next generation of big data storage. Scientists are exploring new technologies and functional materials to surpass the storage capacity constraints of optical discs. Volume holographic storage technology can record two-dimensional information data pages in three-dimensional space, and it has the characteristics of a large capacity and a high writing/reading speed, which is considered to be a very suitable storage method for cold data [1,4,5,6,7]. Photopolymers have attracted considerable attention owing to their application potential. They utilize irreversible photo-initiated polymerization reactions to achieve information recording, with a high resolution, good optical performance, and other advantages. Thus, they are currently a kind of ideal volume holographic material compared to other optical materials [8,9,10,11]. Phenanthrenequinone-doped poly (methyl methacrylate) (PQ/PMMA) is considered as a promising holographic storage photopolymer [12]. This is because its preparation method is simple, and it can also be used as a storage medium with a certain optical storage capacity [13,14,15]. An in-depth study has been performed on the photophysical and photochemical processes of PQ/PMMA polymers. The photoreaction process of PQ is usually required to induce its interactions with electron-donating groups [12,16]. In this system, PQ reacts more readily with MMA and undergoes a [4 + 2] cycloaddition reaction between C=O and C=C [16,17,18,19].

However, there are some disadvantages of single monomer polymerization for PQ/PMMA, such as poor photosensitivity, insufficient diffraction efficiency, etc. These limit the information recording speed of holographic storage [4,5,6]. To increase the photoreceptor speed of the material, improving the exposed sensitivity, one method is to introduce other copolymer monomers with specific properties to modify the substrate [1,12,20]. Dendrimers are also one of the choices in copolymer monomers, with their ability to form crosslinked macromolecules. They exhibit a tight architecture, symmetrical structures, high branching, nanoscale sizes, and delicate three-dimensional structures. Further, their molecular size, shape, and volume can be finely tuned, and the regularity and homogeneity of the structure make the relationship between the structure and properties of dendritic macromolecules more reasonable. The kind and number of functional groups of the dendrimer nuclei, and branched units determine the shape and size of the dendrimers, which have different functions [21,22,23]. The introduction of a dendritic crosslinking agent to modify the polymer to form a dense three-dimensional crosslinked network structure can improve the rigidity and strength of the polymer substrate. Flory first theoretically demonstrated the potential role of branched units in the construction of macromolecules [24]. With the continuous development of synthesis methods, research studies on dendrimers have surged; in recent years, the research focus has gradually shifted from synthesis and property analyses to functionalization and application. Current research is focused on applying the potential properties of dendrimers to different fields such as organic chemistry, analytical chemistry, biology, medicine, materials science, pharmacology, agrochemistry, environmental chemistry, and so on [23,25]. In addition, it plays a role in optical performance improvement [26,27]. Therefore, the main purpose of this study is to modify the substrates of photopolymers by introducing dendritic reactive monomers containing double bonds.

Pentaerythritol is an important chemical raw material with four hydroxyl functional groups in its molecular structure, which is spatially tetragonal and structurally symmetrical. It is very suitable for the construction of dendrimers and therefore plays an important role in the synthesis of dendrimers [28]. Since the emergence of pentaerythritol-based dendrimers in the late 1980s, a variety of pentaerythritol-based dendrimers have been synthesized, which can be widely used in the production of coatings, paints, explosives, plasticizers, nonsurfactants, pharmaceuticals, and pesticides [28]. In this study, a pentaerythritol-based dendrimer with four acrylic groups was used for modification according to the photochemical reaction mechanism of the PQ/PMMA system.

Pentaerythritol tetraacrylate (PETA), a clear, colorless liquid with a characteristic mild odor, is a tetrafunctional acrylate monomer that can polymerize to form crosslinked polymers with a high crosslinking density [29,30,31]. PETA is used as a reactive diluent in UV-curable coatings, inks, and adhesives and also as a crosslinking agent for various polymers.

Herein, a modification was carried out by introducing PETA, a dendrimer, and the properties of the substrate polymer improved compared with those of the conventional PQ/PMMA material. This property improvement verified the success of the modification. A bubble-free photopolymer with a high sensitivity (~2 times higher than that of the conventional PQ/PMMA material) and a short response time was obtained. Moreover, the introduction of PETA allows the grating diffraction efficiency of this system to reach ~80%, and the holographic performance improves. It can also save time in material preparation, unlike previous preparation methods. In this study, the results of the characterization tests illustrated the introduction of more C=C double bonds, and it also indicated that the photoreaction does react in the same way. Moreover, the addition of PETA effectively increased the T_g_ (the glass transition temperature) of the base polymer, and the occurrence of modification was verified; this simultaneously raised the upper limit of the temperature at which engineering plastics can be used. In addition to the successful manufacturing of the PQ/PETA-PMMA photopolymers with potential for holographic storage applications, the density functional theory calculation results elucidate the activation energy decreased by ~0.5 KJ/mol and the excited process of PQ during the photoreaction stage.

## 2. Materials and Methods

**Materials.** Monomer methyl methacrylate (MMA, 99.5%), photosensitizer phenanthraquinone (PQ, 99.0%), and thermal initiator 2,2-azobis(2-methylpropionitrile) (AIBN, 99.9%) were obtained from Shanghai Macklin Co. Ltd., Shanghai, China. Comonomer pentaerythritol tetraacrylate (PETA) was obtained from Aldrich. Dimethyl sulfoxide (DMSO, gas chromatography) was purchased from Shanghai Aladdin Biochemical Technology Co. Ltd., Shanghai, China.

**Figure 1:**
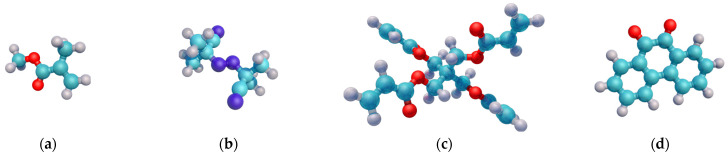
(**a**) Methyl methacrylate space structure (red: O atom; cyan: C atom; gray: H atom). (**b**) 2,2-azobis(2-methylpropionitrile) space structure (purple: N atom; cyan: C atom; gray: H atom). (**c**) Pentaerythritol tetraacrylate space structure (red: O atom; cyan: C atom; gray: H atom). (**d**) Phenanthraquinone space structure (red: O atom; cyan: C atom; gray: H atom).

**Sample preparation.** First, the materials were prepared using the method in Figure S1, based on previously reported preparation methods [17,32,33,34,35,36]. In this process, 100 wt% of the monomer MMA was used for the substrate preparation; AIBN, where the weight used was based on MMA 1%, was used for free radical initiation; and the crosslinking agent PETA was blended, where the weight used was based on MMA 10%; while PQ, where the weight used was based on MMA 1%, for photoreactivity was added for grating initiation before curing. The spatial structural formulas of all the compounds are shown in Figure 1a–c. To investigate the effect of PETA addition on the macroscopic phenomena of the holographic material, the amount added was varied, such as 5, 10, 15, and 20 wt%, all based on the MMA as an example. The specific arrangement is shown in Table 1.

**Figure 2:**
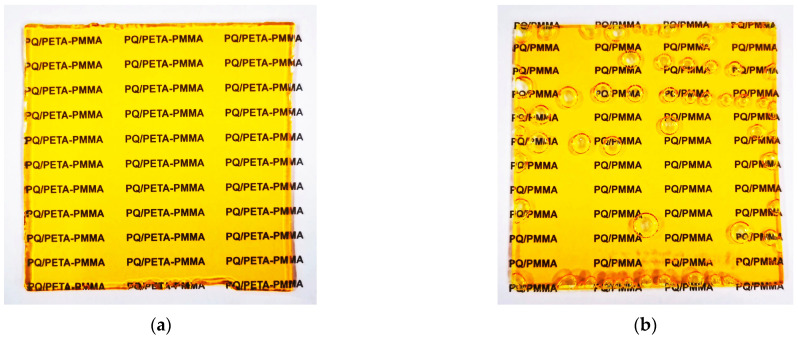
(**a**) PQ/PETA-PMMA molding case (2 h baking). (**b**) PQ/PMMA molding case (20 h baking).

The weighed mixture of each reagent was added to a 30 mL sample bottle to facilitate subsequent processing. Then, it was followed by ultrasonic dissolution and water bath mixing. Unlike previous work, in this study, the mixing time was strictly controlled because of the high activity of the crosslinker; otherwise, the gel point would be rapidly breached, resulting in the material being jellified. Usually, a 333 K water bath was used to sonicate for 5 min, followed by a 333 K water bath with magnetic stirring for half an hour for thorough mixing and preheating. The mixed clear liquid was then injected into a pre-prepared abrasive made of two pieces of glass sandwiched together, in which the center frame was made of polytetrafluoroethylene material with a thickness of 0.15 cm. In the next step, the molded samples were placed in an oven at 333 K for curing, in conjunction with the properties of the crosslinking agent PETA, and the same intervals were taken for the curing times, e.g., 2, 8, 14, and 20 h. In this study, we first evaluated the effect of the curing time. After choosing a suitable curing time, we examined the effect of the crosslinker content. At the end of the baking process, the molds were removed and demolded to obtain the holographic material for recording. The method of preparation of the conventional material used for comparison was the same as that reported in the literature [5,6,37]. The final molded sample is shown in Figure 2a,b. Evidently, the modified PQ/PETA-PMMA material has fewer bubbles, where the bubbles usually emerge because of the negative pressure of the outside air [38]. A crosslinking molding material can effectively isolate air exchange to reduce the air bubbles formed due to its fast molding time. Second, the samples used to study the photoresponses were prepared using a noncuring scheme, in which the monomer MMA and crosslinker PETA were each dissolved individually with PQ, and the exposure experiments were carried out after thorough stirring in a water bath. A portion of the sample was also taken and dissolved in DMSO solvent for subsequent experiments on excitation velocity determination. Third, the samples used to verify the substrate modification were cured using a solution without PQ. But, because it had a blocking effect, the subsequent mixing time was halved to maintain the fluidity of the solution and to prevent gelation. The cured samples were ground into a powder and set aside.

**Holographic performance testing.** The optical path used in the experiment is shown in Figure S2. Two beams of coherent light were chosen to be recorded interferometrically at an angle of 24° symmetrically. The one-way beam was blocked when reading diffraction, the method of recording unslanted volume transmission hologram was adopted in this study. The light source used was a 532 nm green laser with a power of 20 mW, and the diaphragm was adjusted to obtain a spot size of 5 mm. The interference time was recorded as 6 s, and the diffraction time was read as 0.5 s. The evaluation index for the holographic performance is the diffraction efficiency η, which is expressed as follows:

(1) $$\eta =\frac{I_{1}}{I_{1}+I_{0}}$$

where $I_{1}$ and $I_{0}$ denote the Bragg-matched grating diffracted light intensity and the light transmitted through the material, and these can be read from the two photodetectors PD2 and PD1, respectively, so that the marking can ignore the absorption and scattering of the light intensity by the material. The sensitization coefficient S of the material can be obtained from the diffraction efficiency that varies with exposure time [39]:

(2) $$S=\frac{1}{Id}\left(\frac{\partial \sqrt{\eta }}{\partial t}\right)$$

where η is the diffraction efficiency, I is the incident light intensity (0.102 W/cm^2^), and d is the material’s thickness (0.15 cm).

**Characterization.** FTIR spectroscopy was used to analyze the changes in functional groups and their contents during the curing process. The samples, which were previously cured and ground into powder, were grouped according to the curing time and the amount of crosslinking agent PETA added and were tested separately. The infrared test platform used was the SHIMADZU Iraffinity-1s, with a set scanning wave number range of 400–3000 cm^−1^ and a resolution of 4 cm^−1^, and the preparation method was KBr compression. Also, a transform infrared spectroscopic analysis of chemical bonds combining functional groups was applied to photoreactions; the PQ and PETA were exposed to a green laser with an intensity of 0.102 W·cm^−2^ for 48 h to obtain fully exposed samples. Raman spectroscopy was also used to validate the generation of photoproducts in the photoreaction, using the OCEANHOOD RMS2000 Micro Raman Spectrometer, with a measurement beam in the range of 600–3000 cm^−1^ and a resolution of 6 cm^−1^. In addition, a METASH UV-vis-5200 spectrophotometer was used to characterize the light reactions and absorption spectra of the materials based on the Lambert–Beer law:

(3) $$A=\mathrm{lg}\left(\frac{1}{T}\right)=\varepsilon bc$$

where A is the absorbance of the sample, T is the transmittance (also called the transmittance ratio as the ratio of the outgoing light intensity $I_{t}$ to the incident light intensity $I_{0}$), ε is the molar absorption coefficient, b is the concentration of absorbance units, and c is the sample thickness. The absorption spectra of the samples were scanned after the exposure test experiments by placing samples predissolved in DMSO in a 1 cm × 1 cm cuvette.

Furthermore, the characterization methods regarding substrate modification used differential scanning calorimeter for determining the change in the polymer *T_g_*. The test platform was METTLER TOLEDO’s STARe system differential scanning calorimetry (DSC), using a test temperature range of 0–250 °C, with a ramp-up rate of 10 K/min, and a ramp-down which was the opposite. Based on the results of the infrared experiments and for the purpose of reducing preparation time, the samples used for testing were powders with different baking times.

**Theoretical Calculations.** Regarding the theoretical calculations in the photoreaction, all molecular models were optimized using Gaussian 09 (B3LYP/6-31G (d, p)) [40,41,42,43,44]. The electrostatic potential maps of each molecule were made by Multiwfn [45,46] computational analysis combined with VMD [47] The excitation analysis about PQ was carried out by TDDFT (B3LYP) [48], and the reason for the reaction site was analyzed in conjunction with Multiwfn to analyze the valence layer electron leaps. Finally, for the reaction process the same generalization and benchmark were also used for transition state search and intrinsic reaction coordinate (IRC) calculations. Because of the focus on relative values, a single point can be calculated to expand the unit to 6-311G (2d, p) [49].

## 3. Results and Discussion

**Figure 3:**
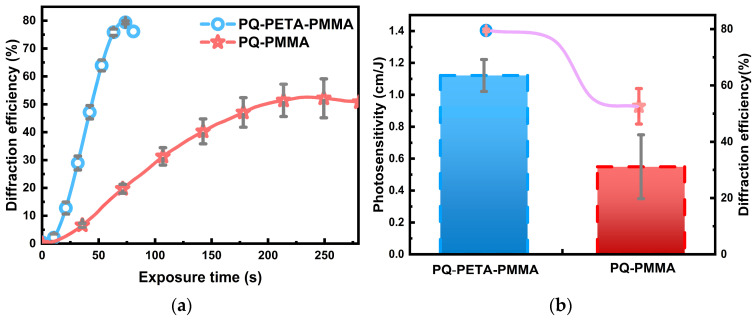
(**a**) Comparison between the diffraction efficiencies of PQ/PETA-PMMA and conventional PQ/PMMA. (**b**) Comparison of the optimal diffraction efficiency and photosensitivity of PQ/PETA-PMMA with those of conventional PQ/PMMA.

**Holographic optical performance of modified material.** Because this study was focused on holographic storage, the macroscopic performance values of the modified material, such as diffraction efficiency and photosensitivity, were evaluated first. In conventional holography, two beams of light with the same vibrational direction, the same frequency, and a constant phase difference are usually used for interference. Benefiting from the light path in the experiment and Equation (1), the result displayed in Figure 3a indicates that the modified PQ/PETA-PMMA material forms a better grating in the recording interference than the conventional material, and the diffraction efficiency in reading can reach ~80%, which is nearly twice that of the conventional PQ/PMMA material. In addition to the increase in the diffraction efficiency as shown in Figure 3a, the speed of formation of the optimal grating (with the highest diffraction efficiency) decreased by a factor of 3.5, from ~250 to ~70 s. Thus, we can also combine Equation (2) to convert the diffraction efficiency over time into photosensitivity (S), a metric that responds to the speed of response of the material’s grating, and the corresponding result is shown in Figure 3b. Evidently, this metric improves from ~0.58 cm/J for the original material to ~1.18 cm/J, which is consistent with the conclusions drawn from the diffraction efficiency (which can increase the speed of optical storage to some extent).

**Figure 4:**
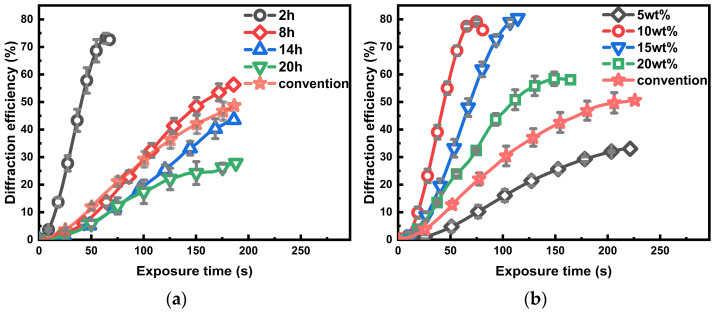
(**a**) Comparison between the diffraction efficiency of the modified and conventional materials with different baking times. (**b**) Comparison between the diffraction efficiency of the modified and conventional materials using different mass percentages of the crosslinking agent.

With the initial modification effect, combined with the desire to reduce the preparation time of the material, further refinement experiments on curing time and crosslinker incorporation were conducted. Figure 4a shows that a baking time of 2 h is suitable for reproducing better results, consistent with the results expected for the activity of the crosslinker PETA. Crosslinked modified materials can already be molded without air bubbles in 2 h, which contributes to increasing the usable area of the material, but it is difficult for conventional PQ/PMMA materials. Next, we examine the mechanism underlying the observed fast molding. The quantitative calculation results are shown in Table S1, which indicates the Gibbs free energy changes in the reactions of PETA + MMA and MMA + MMA. Evidently, the ΔG of the former is negative, which suggests that this group of reactants can react faster, thus aiding in molding. After fixing the baking time and then varying the amount of the added crosslinker, we found that 10 wt% is a more desirable amount, and the results are shown in Figure 4b. The reasons for the determination of these two sets of parameters are explained in the subsequent microchemical mechanism analysis.

**Figure 5:**
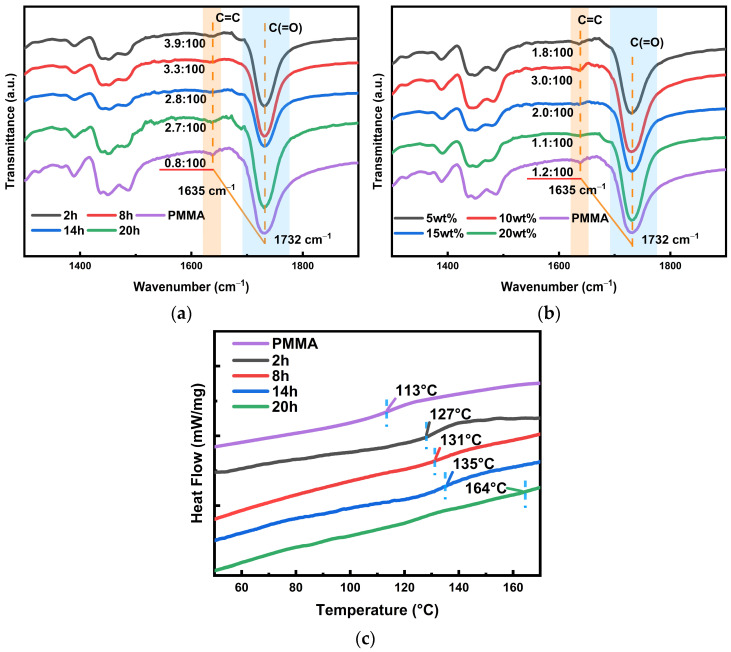
(**a**) FTIR spectra of the modified materials with different baking times. (**b**) FTIR spectra of the crosslinkers using different mass percentages of modified materials. (**c**) DSC curves of the modified materials with different curing times.

**Chemical mechanism analysis through infrared spectroscopy of modified PETA-PMMA base material.** The material reactions are divided into thermal polymerization and photopolymerization. We first gain an understanding of the thermal reaction process by microanalysis using infrared spectroscopy. Infrared spectroscopy allows the analysis and identification of molecules of a substance. A beam of infrared rays of different wavelengths is irradiated onto the molecules of the substance, and the molecules continue to vibrate and rotate, resulting in changes in the transmittance, and some specific wavelengths of infrared rays are absorbed to form the infrared absorption spectrum of the molecule. According to the experimental part of the characterization test, the modified materials are divided into two groups: curing time and concentration. Figure 5a shows the spectral profiles of different curing times; we mainly concentrated on the change in C=C double bond at 1635 cm^−1^, which can be initially analyzed as showing that the polymerization of PETA and MMA is an addition of carbon–carbon double bonds. Then, the change in double-bond content with baking time needs to be quantitatively analyzed to analyze the crosslinking of the dendritic macromolecules. In addition, double bonds are involved in the photoreaction process, and more reactants must lead to a fast reaction rate; this will facilitate subsequent holographic recording. Although the signal intensity of the infrared spectra cannot be directly compared quantitatively, the ratio of the changed amount to the unchanged amount is used to pinpoint the change in the double bond. Because C(=O) (peak at 1732 cm^−1^) is the group in the middle of the molecular chain, and the polymerization reaction occurs at the end group, its content does not change during the thermal reaction. The ratio case is marked in Figure 5a, which indicates that the content of double bonds decreases from ~3.9% to ~2.7% with increasing curing time. Therefore, we can conclude that prolonged baking time increases the crosslinking density, i.e., PETA binds more MMA. Simultaneously, its content is nearly five times higher than that of traditional PMMA substrates. To take a grafting case that can combine the holographic property improvement, combined with the diffraction efficiency shown in Figure 4a, an optimal curing time of 2 h is determined. The infrared profiles grouped by concentration changes in Figure 5b indicate that the content of the double bonds also first increases and then decreases with the increasing PETA content. However, in this group, the double-bond content in each sample is lower than that in the group with varying curing times; only the 10 wt% PETA content is close. Similarly, the diffraction efficiency in Figure 4b again verifies the feasibility of the 10 wt%, proving that a 10 wt% content is relatively reasonable. Because holographic recording requires a certain amount of double bonds, i.e., reactants for light reactions, the situation of concentration change will not be discussed below.

This section describes the analysis of crosslinking material modification. The occurrence of crosslinking modification can already be preliminarily predicted from the infrared spectra of different curing times in thermal reactions; to continue to analyze the modification of polymer substrates further, we test the change in glass transition temperature. The glass transition temperature (T_g_) is the temperature corresponding to the transition from the glassy state to the elastic state. The glass transition is an inherent property of amorphous polymer materials and is a macroscopic manifestation of the transformation of the form of polymer motion, which directly affects the usability and process performance of the material; therefore, in this study, we investigate the change in T_g_ under different baking times, so as to prove the existence of dendritic modification. The selected assay method is DSC curve analysis. Figure 5c shows that the T_g_ of the conventional substrate is 113 °C. There is a significant increase in T_g_ with the increasing baking time of the material and addition of PETA (10 wt%), which confirms the dendritic macromolecule modification. The complete DSC curve of heat absorption and excretion is Supplemented in Figure S3.

**Figure 6:**
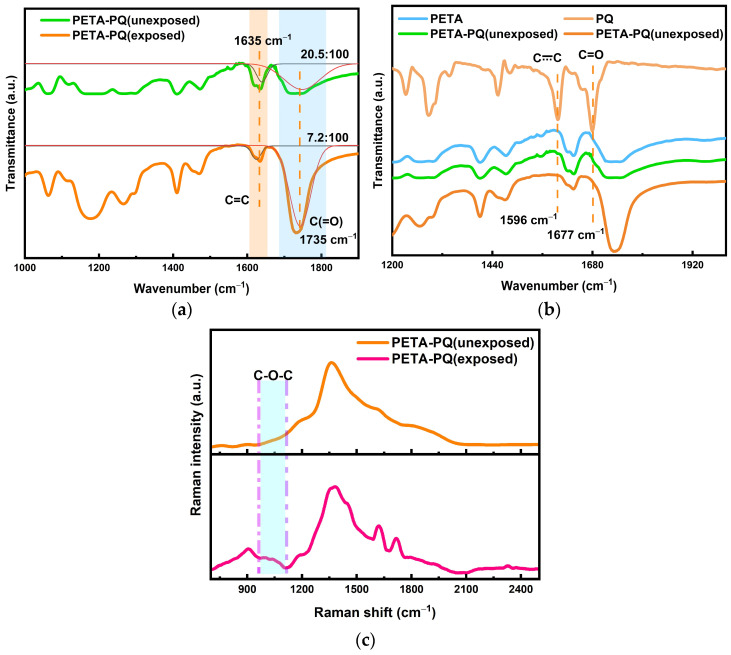
(**a**) FTIR spectra of double-bond changes during the PETA and PQ photoreactions. (**b**) Infrared spectra showing the functional group changes in PETA and PQ during the photoreaction of PETA with PQ. (**c**) Raman spectra of PETA and PQ indicating the formation of new bonds.

**Overall analysis of the photoreaction process.** The infrared spectroscopic results of the thermal reaction process tentatively indicate an increase in the amount of reactants that can be made available for holographic recording, and this is followed by a microanalysis of the material exposure process, i.e., the holographic recording stage, from the point of view of the photoreaction. First, infrared spectroscopy is employed to determine the already prepared photoreactive uncured samples, and the results indicate a decrease in the double-bond content in the exposed samples, as shown in Figure 6a (the plot is fitted), from ~20.5% to ~7.2%. This decrease in content demonstrates that a [4 + 2] addition reaction may occur after the exposure. In conjunction with Figure 6b, although the blue PETA alone has a strong infrared spectral signal that masks the signal of the PQ, it Is still possible to see two peaks of PQ Itself appearing In the pre-exposure sample, the vibration of the benzene ring itself at 1596 cm^−1^ and the C=O carbonyl vibration at 1677 cm^−1^. Further, there was a disappearance of the signals of these two peaks after the exposure, which again illustrates the [4 + 2] addition, i.e., the addition of the PQ carbonyl to the PETA double bonds. To determine the generation of C-O-C bonds in this reaction, Raman spectra of the samples were analyzed. Because the spectral bands of Raman activity reflect the change in group polarizability with simple positive vibrations, and the infrared spectra also reflect the change in the dipole moment of a group with simple positive vibrations, the Raman spectra contain fewer multiplicative and group spectral bands than those in the infrared spectra. This implies that Raman spectroscopy can be used to analyze the groups that cannot be evaluated using infrared spectroscopy. The analysis result is shown in Figure 6c; evidently, the peak of C-O-C appears at 1000 to 1100 cm^−1^, which is basically consistent with the results reported in the literature [1,17,32]. Up to this point it has been shown experimentally that PETA, like MMA, can photoreact with PQ, which provides a priori conditions for the subsequent theoretical calculations of the results.

**Figure 7:**
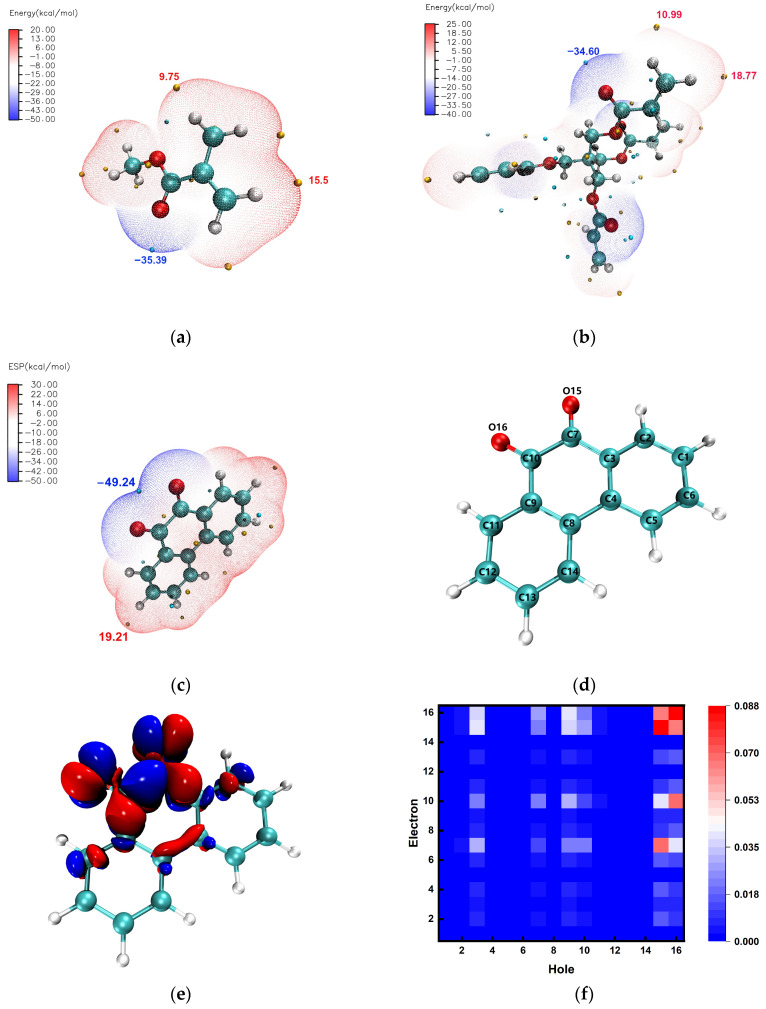

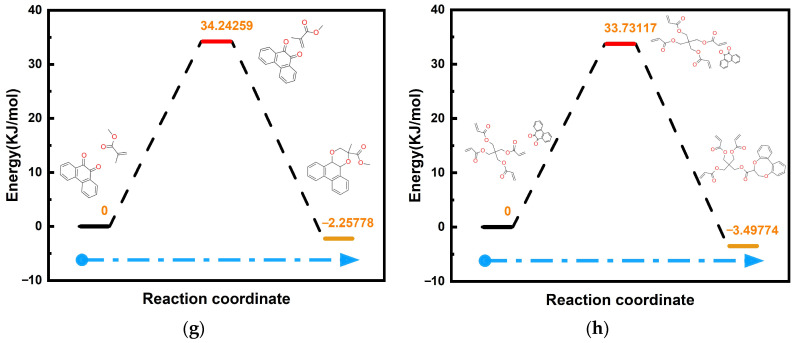
(**a**) Molecular surface electrostatic potential and extreme points of MMA. (**b**) Molecular surface electrostatic potential and extreme points of PETA. (**c**) Molecular surface electrostatic potential and extreme points of PQ. (**d**) Number of each atom in the PQ molecule. (**e**) PQ electron jump density (S_0_ to S_1_, isovalue: 0.01 a.u.). (**f**) PQ TDM (S_0_ to S_1_). (**g**) Energy of the PQ reaction with MMA. (**h**) Energy of the PQ reaction with PETA.

To investigate the reaction rate deeper, the noncovalent bonding effects were theoretically analyzed. Different extramolecular arrangements of the electron cloud will lead to the molecular surface showing a different potential difference; this will generate electrostatic potential. For the examination of intermolecular electrostatic interactions, predicting reaction sites, and predicting the nature of the molecule, this has an important significance [50,51]. Further, the molecular surface electrostatic potential map by the size of the electrostatic potential of the different surface areas through the different colors shows that the molecular surface of the distribution of the potential of the electrostatic force is clear at a glance [52]. Then, with the help of theoretical calculations and wave function analysis, we can examine the molecular van der Waals surface maps colored by the electrostatic potential of the molecules involved in the photoreaction. The surface electrostatic potential maps and extreme points of MMA, PETA, and PQ are shown in Figure 7a–c. Evidently, the largest negative point of the PQ molecule is located on the two carbon groups C=O, which according to Coulomb attraction inevitably combine with the positively charged portion. By contrast, MMA and PETA exhibit the same positive electrical properties and are concentrated on the C=C double bonds. However, PETA has more positive points, and thus has a high probability of reaction, resulting in a high rate of photoreaction. Combined with the theoretical calculation of the method of the photosensitizer (PQ), excitation was analyzed, scanning the changes of the 10 excited states shown in Table S2. The results can be seen in the energy of the leap and orbital contribution. The green light used for holographic storage in this study basically only excites the PQ to S_1_ (single heavy state first excited state). In addition, with the help of Multiwfn (version 3.8) wavefunction analysis software, density matrix analysis can be carried out for PQ transitions. Transition matrix is a general term for a variety of matrices that contain information about the characteristics of electron transitions, and the most commonly studied matrix is the transition density matrix (TDM). Figure 7e shows the change in electron density from the S_0_ jump to S_1_ of PQ. It appears as a localized jump near the carbonyl group, which again explains the reason for the reaction site in the photoreaction. Combined with Figure 7d,f, based on the labeling of the individual atoms of the PQ molecule, we can see in the TDM thermogram that the electron–hole distribution is just above the 15^th^ and 16^th^ oxygen atoms. In addition to the analysis of noncovalent bonding interactions, a direct transition state search for the reaction of PQ with PETA and MMA reveals that the reaction energy barrier, i.e., the energy required for the reaction, in PQ + PETA in Figure 7h, is 33.73 kcal/mol. This value is slightly lower than that required for PQ + MMA in Figure 7g. Thus, faster reactions increase the photoreceptor sensitivity in holographic performance, while faster binding assists in rapid molecular diffusion [53,54,55], which can somewhat improve the diffraction efficiency.

**Figure 8:**
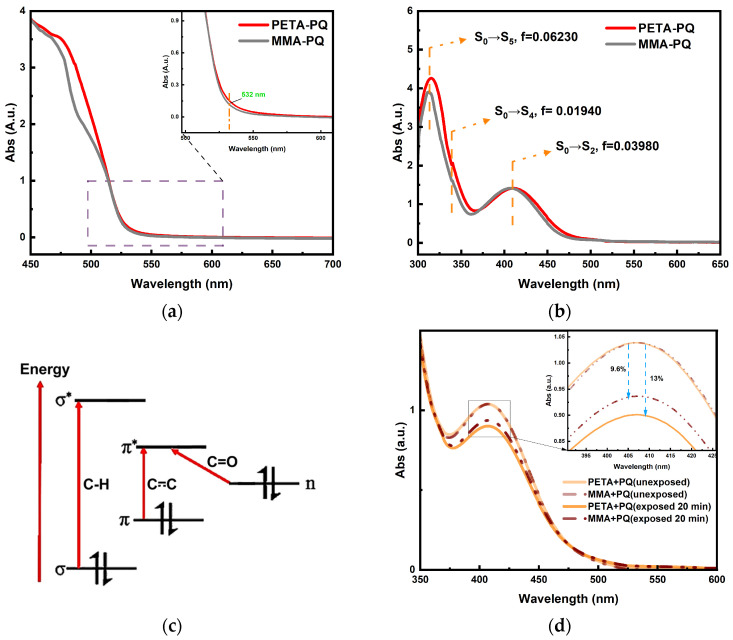
(**a**) Absorption of sheet-modified materials versus those of conventional materials. (**b**) UV–Vis absorption spectrum of PQ. (**c**) PQ electron excitation model. (**d**) Comparison of light reaction rates in the experiments.

Finally, the results of the theoretical calculations are again verified experimentally with the help of a UV–Vis spectrophotometer. Considering the long recording time required in holographic storage, fully exciting PQ is not necessary, thus allowing the complete consumption of the photosensitizer. The overall scanning results of the PQ/PETA-PMMA material are depicted in Figure 8a. The absorption peak at 532 nm is weak and is thus compatible with the requirements of holographic materials. Subsequently, the excitation rate is analyzed and compared with the reaction rate using a pre-prepared sample dissolved in the DMSO solvent. Figure 8b shows three absorption peaks, located at 310, 330, and 419 nm, which correspond to the positions of the large oscillator strength in the theoretical calculations, and the effect of substrate absorption in Figure S5 is ruled out. Corresponding to the electron leaps from σ bonding orbitals to σ* antibonding orbitals at 310 nm, π orbitals to π* orbitals at 330 nm, and n orbitals to π* at 419 nm, the formation of leaps is shown in Figure 8c. This result is in good agreement with those obtained by theoretical calculations. To compare the reaction rate according to Equation (3), we selected samples with the same absorbance, to ensure the same molar concentration of PQ, and the same exposure times. Figure 8d shows that the consumption rate of PQ under PETA is faster than that of MMA, with a drop of ~13%. In contrast, the concentration of PQ only drops by ~9% under MMA, which is not a large decrease but verifies the slight difference in the reaction energy barriers observed in the theoretical calculations.

Finally, the modified material increases by ~5 times in substrate C=C content compared to the conventional material, while the newly introduced PETA is able to react with PQ when espoused, and the reaction energy barrier is reduced by 0.5 KJ/mol. These demonstrate the improvement of the photosensitivity of the material. At the same time, Table 2 lists the properties of some holographic materials to compare the differences between diffraction efficiency and photosensitivity. Although the sensitivity of the PQ/PETA-PMMA material is not the best, it is also a good choice for holographic storage materials combined with diffraction efficiency.

## 4. Conclusions

We demonstrate that the sensitivity of the PQ/PETA-PMMA photopolymer can be significantly enhanced, and the photosensitivity (~1.18 cm/J) increased by ~2 times. The experimental characterization revealed that the modified material can elevate the reactant content, and the theoretical calculation results show the lowering of the photoreaction barrier. At the same time, the PQ/PETA-PMMA photopolymer also has a higher diffraction efficiency (~80%), while the molding and preparation times of the material are considerably shortened, and there are no bubbles, which improves the usable area of the material. In addition to the macromolecular network formed via crosslinking of the dendritic core molecule PETA, we modified the *T_g_* of substrate increased by ~10 °C. Further, quantum chemical calculations unveiled the mechanism of the photoreaction process of the system. In this study, both theoretical calculations and experiments were utilized to jointly investigate the microscopic mechanism of modified photopolymers. Our results indicate that dendritic crosslinking-modified holographic materials will promote the development of new efficient storage materials in the future, suggesting the possibility of holographic data storge industrialization.

## Figures and Tables

**Table 1:** The concentration ratio of each component in the prepared sample.

Concentration (wt%)	MMA(g)	AIBN(g)	PQ(g)	PETA(g)
0	20.00	0.20	0.20	0
5				1.00
10				2.00
15				3.00
20				4.00

**Table 2:** Comparison of the properties of different holographic materials.

Material	*d* (mm)	*S* (cm/J)	*η* (%)
POSS-PQ/PMMA [1]	1.5	1.47	75
Irgacure 784/PMMA [6]	1.5	0.571	52
THMFA [33]	1.5	0.035	95
C 60-PQ/PMMA [34]	1.5	0.59	72
Al NP-PQ/PMMA [37]	1.5	1.54	57
PQ/PETA-PMMA	1.5	1.18	80

## Data Availability

Data are contained within the article and Supplementary Materials.

## Supplementary Materials

The following supporting information can be downloaded at: https://www.mdpi.com/article/10.3390/polym16111484/s1, Figure S1: Preparation process steps; Figure S2: Optical response and holographic performance (diffraction efficiency) test light paths, in which an unslanted volume transmission hologram was used, HWP: halfwave plate; M: reflective mirror; PBS: polarization beam splitter; PD: photo detector; Figure S3: DSC curves on the baking time of PETA-PMMA; Figure S4: Infrared spectrograms of PETA-PMMA with respect to baking time and weight percent; Figure S5: Excitation uptake by the substrate and the solvent itself; Table S1: Free energy changes in thermal reactions; Table S2: Theoretical calculations of ten excited states of the PQ molecule.
