# Supplementary material for: Sensitivity-Enhancing Modified Holographic Photopolymer of PQ/PMMA
Source: Polymers (Basel). 2024 May 23;16(11):1484. doi: 10.3390/polym16111484 (PMC11174760; doi:10.3390/polym16111484)
Supplement: Supplementary file 1 [file polymers-16-01484-s001.zip › polymers-2989534-supplementary.pdf]

Supporting Information

Sensitivity-enhancing modified holographic photopolymer of PQ/PMMA

Junhui Wu†, Junchao Jin†, Po Hu§, Jinhong Li†, Zeyi Zeng†, Jie Liu†, Qingdong Li†, Mingyong Chen†, Zuoyu Zhang†, Xiao Lin*,‡, and Xiaodi Tan*,‡

†College of Photonic and Electronic Engineer, Fujian Normal University, Fuzhou 350117, China
§Henan Provincial Key Laboratory of intelligent lighting, Huanghuai University, Zhumadian 463000, China
‡Information Photonics Research Center, Key Laboratory of Optoelectronic Science and for Medicine of Ministry of Education, Fujian Provincial Key Laboratory of Photonics Technology, Fujian Provincial Engineering Technology Research Center of Photoelectric Sensing Application, Fujian Normal University, Fuzhou 350117, China
*Email: xiaolin@fjnu.edu.cn; Email: xtan@fjnu.edu.cn

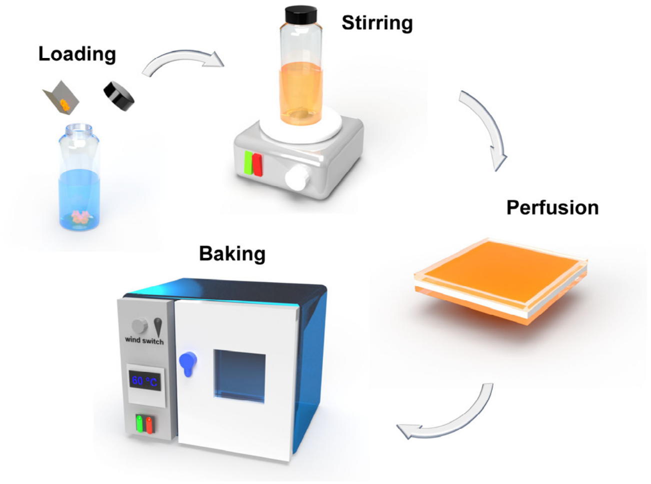

Figure S1. Preparation process steps.

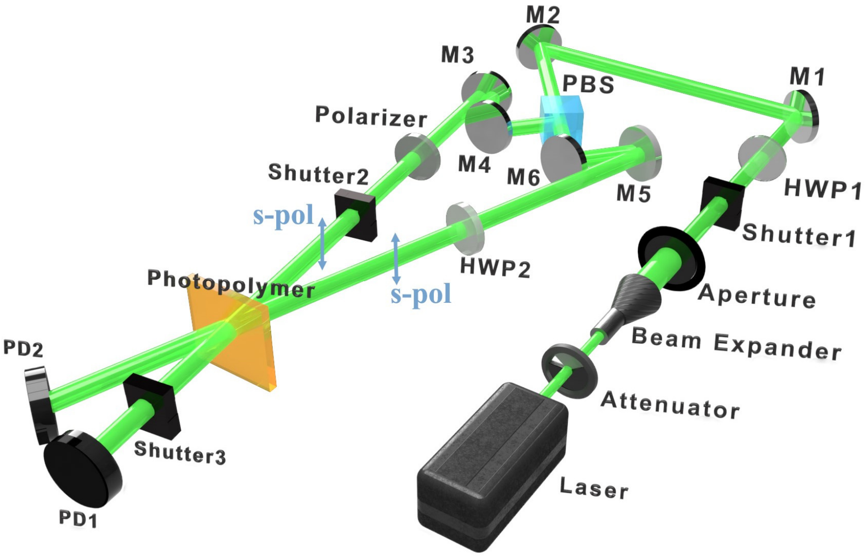

Figure S2. Optical response and holographic performance (diffraction efficiency) test light paths, in which an unslanted volume transmission hologram was used, HWP: halfwave plate; M: reflective mirror; PBS: polarization beam splitter; PD: photo detector.

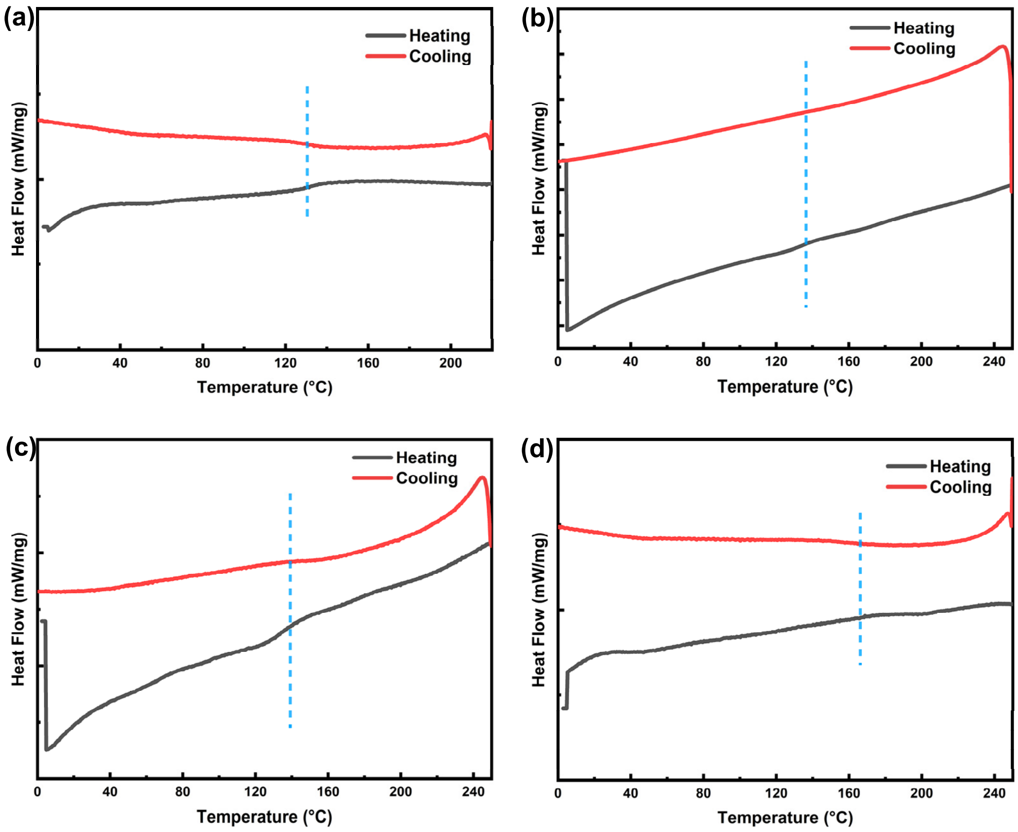

Figure S3. DSC curves on baking time of PETA-PMMA (a) 2h. (b) 8h. (c) 14h. (d) 20h.

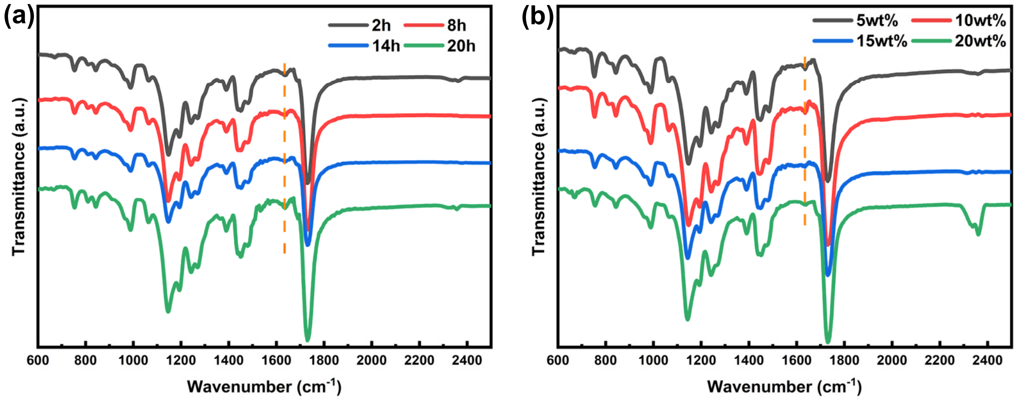

Figure S4. Infrared spectrograms of the PETA-PMMA with respect to (a) baking time and (b) weight percent.

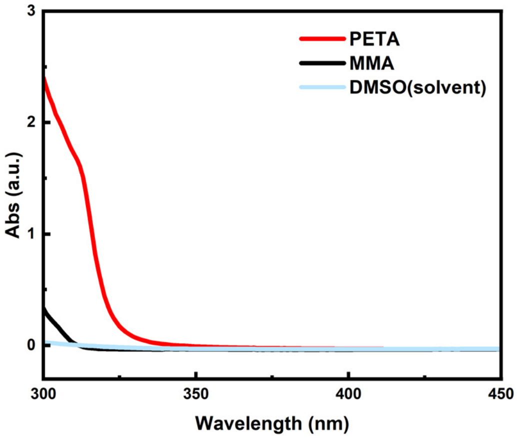

Figure S5. Excitation uptake by the substrate and the solvent itself.

Table S1. Free energy changes in thermal reactions.

Thermal reaction object	Reactant electron energy (hartree)	Generator electron energy (Hartree)	Reactant free energy correction (Hartree)	Generator free energy correction (Hartree)	Gibbs free energy(ΔG，kcal/mol)
PETA+MMA	-1607.8659	-1607.8807	0.3938	0.4069	-1.0429
MMA+MMA	-691.7901	-691.8003	0.1961	0.2079	1.0316

Table S2. Theoretical calculations of ten excited states of the PQ molecule.

Excited state	Energy	Wavelength	Oscillator strength	Orbital contribution
# 1	2.2622 eV	548.07 nm	f= 0.00000	H -> L 98.1%
# 2	2.9697 eV	417.50 nm	f= 0.04110	H-1 -> L 98.7%
# 3	3.4383 eV	360.60 nm	f= 0.00000	H-4 -> L 93.8%
# 4	3.7434 eV	331.21 nm	f= 0.02180	H-2 -> L 94.1%
# 5	3.9908 eV	310.68 nm	f= 0.06630	H-3 -> L 90.5%, H-1 -> L+2 5.6%
# 6	4.4626 eV	277.83 nm	f= 0.00010	H -> L+1 98.5%
# 7	4.6528 eV	266.47 nm	f= 0.00000	H -> L+2 94.6%
# 8	4.7106 eV	263.20 nm	f= 0.01740	H-5 -> L 89.9%, H-1 -> L+2 6.7%
# 9	4.9340 eV	251.29 nm	f= 0.41870	H-1 -> L+1 85.7%
# 10	4.9600 eV	249.97 nm	f= 0.07740	H-1 -> L+2 60.8%, H-3 -> L+1 28.2%, H-3 -> L 5.4%
